# Draft Genome of a Member of the Family *Chromobacteriaceae* Isolated from Anopheles Mosquitoes in West Africa

**DOI:** 10.1128/mra.00524-22

**Published:** 2022-09-19

**Authors:** Keenan Stephens, Edounou Jacques Gnambani, Etienne Bilgo, Abdoulaye Diabate, Scott Soby

**Affiliations:** a Biomedical Sciences, College of Graduate Studies, Midwestern University, Glendale, Arizona; b Institut de Recherche en Science de la Santé (IRSS), Bobo-Dioulasso, Burkina Faso; c College of Veterinary Medicine, Midwestern University, Glendale, Arizona; Loyola University Chicago

## Abstract

Chromobacterium sp. strain IRSSSOUMB001 with potent insecticidal activity was isolated from Anopheles gambiae s.l. in Burkina Faso. The draft genome is 5,090,822 bp and encodes predicted genes for hydrogen cyanide production, haemolysin, a T3SS, and *yopE*, which are potential virulence factors against mosquitoes.

## ANNOUNCEMENT

Control of vector-transmissible diseases such as malaria and dengue depends on effective mosquito population management and reduction. Concerns about environmental damage and the emerging prevalence of insecticide resistance has intensified the search for alternatives to chemical insecticides for controlling mosquitoes, including engineered gene drives (1, 2), wMelPop Wolbachia-induced sterility (3, 4), and the distribution of insecticidal bacteria and fungi or their products into mosquito breeding grounds (5–8). In this study, bacteria were isolated from larvae and the cuticles of adult A. gambiae s.l. mosquitoes in Soumousso, Burkina Faso (11°04′ N, 4°03′ W). A. gambiae tissue homogenates were plated and colony purified on chocolate and polyvitek agars supplemented with bromocresol purple. Chromobacterium sp. strain IRSSSOUMB001 was found to have potent mosquitocidal activity (8). Chromobacterium sp. strain IRSSSOUMB001 was then colony purified three times on King’s medium B (KMB) agar (9), grown overnight in KMB broth for genomic DNA extraction with a DNeasy blood and tissue kit (Qiagen), and provisionally placed within the genus Chromobacterium by 16S rRNA gene sequences that had been amplified with 27F and 1525R primers by BLAST comparison with the NCBI nucleotide database (10). Illumina-compatible libraries were generated by enzymatically shearing gDNA to ≈500-bp fragments, repairing the DNA ends, and adding A-tails (Kapa Biosystem Hyperplus library preparation kit, KK8514). Illumina-compatible adapters (IDT catalog number 00989130v2) were individually ligated to each sample. Ligated molecules were cleaned using Kapa pure beads and then amplified with HIFI enzyme (Kapa Biosciences, KK8002 and KK2502). Each library was sized with an Agilent TapeStation, quantified by quantitative PCR (qPCR) (KAPA Library quantification kit, KK4835), pooled, and sequenced on an Illumina MiSeq platform 2 × 250 flow cell. Assembly, quality control, and annotation were performed using the PATRIC comprehensive genome analysis pipeline version 2.6.12 (http://patricbrc.org) (11) with default settings, except with the trim setting as “true,” which removed barcodes and provided quality control with Trim Galore version 0.4.0 and QUAST version 5.1 (12, 13). A total of 1,634,067 raw reads were assembled into 68 contigs totaling 5,090,822 bp using Unicycler version 0.4.8 and then polished with Pilon version 1.23 (14, 15). GC content was 62.98%, and *N*_50_ was 252,994 bp with 146-fold coverage. The isolate was definitively placed in the genus Chromobacterium using the genome sequence by the Type (Strain) Genome Server (16). The isolate was most closely related to Chromobacterium haemolyticum DSM 19808 (JONK00000000) with a digital DNA-DNA hybridization (dDDH) (d4) of 66.4%. The genome was annotated by RASTtk version 1.073 (17). RAST annotation indicates that several potential virulence effectors against mosquitoes are present in the genome, including genes for hydrogen cyanide (*hcnABC*) (18, 19), phospholipase haemolysin, and type III secretion systems, including a homolog of the versatile GTPase-activating protein effector *yopE* from Yersinia pestis that acts as both a cytotoxin and a suppressor of cellular immune responses (20).

### Data availability.

This whole-genome shotgun project has been deposited at DDBJ/EMBL/GenBank under BioProject PRJNA816544, BioSample SAMN26680783 under the accession number JALCYU000000000. The version described in this paper is version JALCYU010000000. SRA accession is accessible as SRR18349793. RASTtk annotations are available under open license at Zenodo (https://zenodo.org/record/6425622#.Yl38G-HMKUk).
